# Raw bioelectrical data and physical performance in track and field athletes: Are there differences between the sexes in the relationship?

**DOI:** 10.1016/j.heliyon.2024.e35754

**Published:** 2024-08-03

**Authors:** Gabriele Mascherini, Matteo Levi Micheli, Sofia Serafini, Claudia Politi, Eva Bianchi, Álex Cebrián-Ponce, Marta Carrasco-Marginet, Pascal Izzicupo

**Affiliations:** a Department of Experimental and Clinical Medicine, University of Florence, 50134, Florence, Italy; b Department of Medicine and Aging Sciences, University “G. D'Annunzio” of Chieti-Pescara, 66100, Chieti, Italy; c INEFC-Barcelona Sports Sciences Research Group, Institut Nacional D'Educació Física de Catalunya (INEFC), University of Barcelona (UB), 08038, Barcelona, Spain

**Keywords:** Phase angle, Levi muscle index, Sprint, Strength, BIVA

## Abstract

**Objectives:**

The study aimed to investigate the relationship between raw bioelectrical data and physical performance in track and field athletes. Specifically, the objectives were to determine: 1) whether a regional bioelectrical impedance approach provides additional insights compared to whole-body analysis, 2) the reliability of the Levi Muscle Index (LMI) in this context, and 3) whether there are differences in these relationships between male and female athletes.

**Design:**

This study utilized a cross-sectional design involving thirty-one female athletes (mean age 21.4 ± 3.8 years) and thirty male athletes (mean age 21.1 ± 2.6 years) from track and field. On a single day, participants underwent whole-body and regional bioelectrical impedance assessments focusing on the lower limbs, alongside strength and speed performance tests.

**Results:**

The study found no significant differences in the relationship between whole-body versus regional bioelectrical impedance and performance tests. Resistance (R) demonstrated an inverse correlation, while phase angle (PhA) and Levi Muscle Index (LMI) showed direct correlations with most performance variables in track and field athletes. Significant differences were observed between male and female athletes across all parameters, with male athletes exhibiting superior performance, higher PhA and LMI values, and stronger correlation coefficients compared to females.

**Conclusions:**

In summary, this study highlights the intricate relationship between body composition and physical performance in athletes. It underscores the importance of considering sex differences and the reliability of raw bioelectrical data, whether obtained through regional or whole-body approaches, in assessing athletic performance.

## 1 Introduction

The study of body composition in sports using bioelectrical impedance analysis (BIA) continues to evolve rapidly [1]. Understanding the relationship between different body compartments and physical performance is of particular interest in this field. Current research in BIA includes the application of impedance vector analysis, known as bioelectrical impedance vector analysis (BIVA) [2]. The BIVA approach utilizes raw bioelectrical data, where impedance (Z) is defined by the relationship between resistance (R) and reactance (Xc), with R representing the opposition to current flow and Xc indicating the delay caused by cell membrane capacitance. A key derived parameter is phase angle (PhA), calculated as the arctangent of Xc/R × 180°/π, typically ranging from 1° to 12° in the human body, which reflects cellular membrane integrity and function [3]. An additional parameter, the Levi Muscle Index (LMI), recently proposed for assessing muscle mass in sports populations, adjusts PhA for variations in body hydration, providing a more specific measure of muscle mass (defined as PhA/(R/height)) [4].

Several studies have demonstrated associations between these raw bioelectrical data and physical performance across various sports disciplines [5]. For instance, R has been negatively correlated with 1) running time in male and female trail runners [6], 2) endurance performance in male soccer players [7], and 3) maximal mean power in professional male cyclists [8]. PhA, on the other hand, has shown positive associations with 1) VO_2_max in male professional futsal players [9], 2) concentric rate of force in alpine skiers [10], and inversely related to 3) sprint times in male youth soccer players [11], and 4) time of 50 m all out in competitive male and female swimmers [12]. In this context, LMI offers potential to enhance understanding by adding muscle mass insights to other raw bioelectrical data in the sports population.

Recently, a regional approach to bioelectrical impedance analysis has emerged, facilitating the assessment of specific body segments. Unlike whole-body analysis, which assumes uniform resistivity across the body, the regional method measures bioelectrical data from distinct areas, potentially providing more detailed insights [13]. In sports, certain body segments are more crucial than others, and studies have shown that regional bioelectrical impedance analysis (BIVA) can offer nuanced information compared to whole-body assessments. Significant changes in BIVA parameters following intense exercise have been observed more distinctly with regional assessments than with whole-body measures [5]. For example, regional BIVA at the lower limbs has proven informative for athletes in football [14], cycling [15], and rowing [16], highlighting sex differences such as higher lower limb PhA values in male footballers compared to females [14]. Moreover, studies, such as those on male cyclists in the 2012 Giro d'Italia, indicate that while whole-body PhA remained unchanged, regional assessments showed a decrease in lower hemisphere PhA over a three-week stage race [15]. In rowers, upper hemisphere PhA has shown greater relevance to performance compared to whole-body PhA assessments [16].

However, despite these advancements, the association between whole-body and regional BIVA and sports performance remains underexplored across various sports disciplines. Therefore, this study aims to enhance understanding of the relationship between raw bioelectrical data and physical performance in male and female track and field athletes. Specifically, the study will evaluate the strength and speed performance of athletes' lower limbs and correlate these findings with whole-body and regional measurements of resistance (R), reactance (Xc), phase angle (PhA), and Levi Muscle Index (LMI). Additionally, the study will investigate potential sex differences in these relationships, providing insights into how body composition influences athletic performance differently between males and females.

## 2 Methods

### 2.1 Participants

This cross-sectional study enrolled 61 Italian track and field athletes. This study followed STROBE guidelines. The inclusion criteria were: 1) age between 18 and 35, 2) registered with the Italian track and field federation for the current season, 3) having practiced track and field at a competitive level for at least ten years, 4) being classified as at least tier 3 athletes: Highly Trained/National Level [17], 5) having had no injuries or surgeries that could affect participation in sports activities in the previous three months, and 6) not taking any medications.

The sample comprised 31 female athletes (21.4 ± 3.8 years, 166.1 ± 6.1 cm, 57.4 ± 9.7 kg) and 30 male athletes (21.1 ± 2.6 years, 180.1 ± 5.0 cm, 72.5 ± 10.5 kg). Specifically, 23 sprinters (12 females and 11 males), 12 throwers (6 females and 6 males), 15 marathon runners (7 females and 8 males), and 11 jumpers (6 females and 5 males) were registered. Therefore, the sex distribution between different disciplines is balanced.

All measurements were conducted at the Luigi Ridolfi Stadium in Florence. Subjects were enrolled after providing written informed consent. The study was carried out in accordance with the ethical standards in the 1975 Declaration of Helsinki. Ethical approval for this study was granted by the Ethics Committee for Clinical Sport Research of Catalonia (Ethical Approval Code: 0022/CEICGC/2023).

### 2.2 Procedures

Recruitment and participant evaluations were conducted during the in-season phase when athletes typically exhibit optimal body composition (i.e., lowest fat mass and highest fat-free mass). All assessments took place in the morning with participants in a fasting state and after voiding their bowels and bladder. Additionally, participants refrained from consuming caffeine, alcohol, and engaging in strenuous exercise on the day preceding the assessments to minimize potential confounding factors.

### 2.3 Body composition assessments

Body composition assessments preceded the performance tests. Body mass was measured to the nearest 0.1 kg and height (H) to the nearest 0.5 cm (Seca GmbH & Co., Hamburg, Germany). Body mass index (BMI) was calculated as body mass divided by height squared (kg/m^2^).

**Fig. 1:**
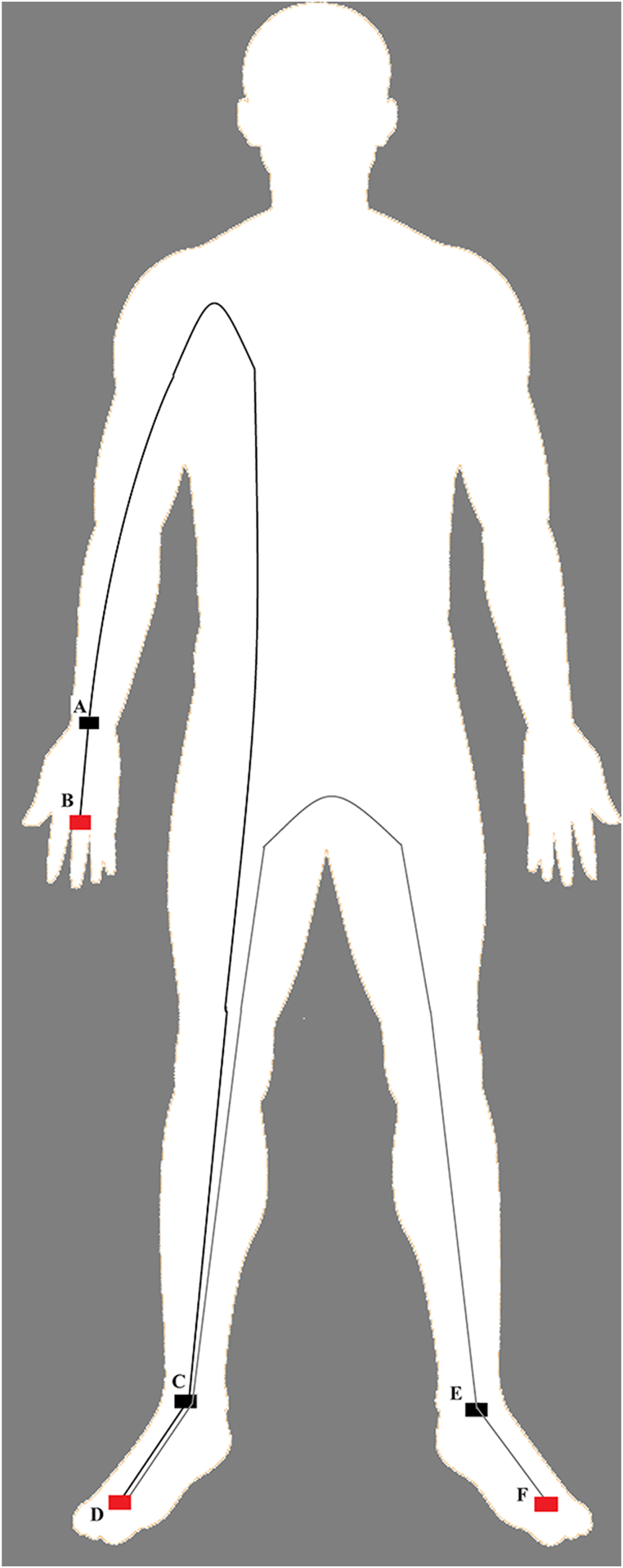
Procedure for performing whole body and regional BIVA. The whole body is conventionally performed on the right side of the body with the injector and detector electrodes on the right hand (A–B) and right foot (C–D). The regional evaluation of the lower limbs occurs with the electrodes between the right foot (C–D) and the left foot (E–F). The two bioelectric circuits are represented, the whole body in black color and the regional for the lower limbs in gray color.

Bioelectrical measurements were obtained using the BIA 101 Anniversary Sport Edition analyzer (Akern, Florence, Italy), which emitted a 400 mA alternating sinusoidal current at 50 kHz (±1 %). Calibration was performed with a known impedance circuit provided by the manufacturer (R = 383 ± 10 Ω, Xc = 45 ± 5 Ω). According to the manufacturer's guidelines, participants were tested with their arms and legs held away from the body, with legs open at 45° to the body's midline and upper limbs positioned 30° away from the trunk. After skin preparation, two electrodes (Biatrodes Akern Srl, Florence, Italy) were placed on the hands and feet on both sides, totaling eight electrodes for each measurement. To minimize electric field interaction, the detector electrodes were positioned approximately 5 cm away from the injector, thereby reducing the risk of overestimating impedance values. Finally, a stabilization period of 5 min preceded the assessment, covering the entire body (hand to foot on the right side) and the lower hemisome (foot to foot), as illustrated in Fig. 1 and previously described [18]. All BIA measurements were consistently performed by the same trained investigator to minimize inter-observer errors and ensure data accuracy and reliability. R and Xc were standardized for subject height to adjust for conductor length (R/H, Xc/H). PhA was defined as tan⁻^1^(Xc/R · 180°/π), and LMI as PhA/(R/height) [4,19].

### 2.4 Performance tests

Following the body composition assessment on the same day, athletes engaged in their customary 15-min warm-up routine. Subsequently, the following performance tests were conducted to evaluate speed and lower limb strength.

- Sprint on 5 and 10 m: Athletes were instructed to run as fast as possible upon a starting signal. Time was measured from the start line to the finish line using double optical photocells Witty Gate (Microgate Srl, Bolzano, Italy).
- Standing long jump: Athletes jumped as far as possible from a standing position, aiming to land with both feet together. Distance was measured in centimeters from the last heel strike to the take-off line.
- Triple Jump: Athletes started from the starting line and performed three consecutive maximal jumps forward, alternating supporting limbs. Distance was measured from the take-off point to the landing.
- Squat Jump: Athletes squatted to 90° knee flexion, maintained the position briefly, and then jumped as high as possible without preparatory movement, hands on hips [20].
- Counter Movement Jump: Athletes performed a downward movement followed by full extension of the hip, knee, and ankle joints, keeping hands on hips.
- Stiffness Jump test: Athletes performed seven stiff-legged pogos, aiming to minimize ground contact [21].

All tests were familiar to the athletes and were conducted under standardized conditions in regarding the sequence and time of day to ensure result accuracy and consistency. Vertical jumps were performed while wearing the BTS G-Walk sensor 2 (BTS Bioengineering, Milan, Italy), a wearable inertial device [22]. Data were transmitted via Bluetooth to a notebook and analyzed using BTS G-Studio software (BTS Bioengineering, Italy). Three measurements with 1 min 30 s of rest between trials were conducted for each test, and the mean value was used for data analysis. The BTS G-Walk sensor 2 provided parameters including take-off force (kN), landing force (kN), maximum concentric power (kW), average speed during concentric phase (m/s), peak speed (m/s), and take-off speed (m/s).

### 2.5 Statistical analysis

Descriptive statistics (mean, standard deviation) were computed for each variable, and the normality of the data was assessed using the Shapiro-Wilk test. The student's unpaired *t*-test was employed to analyze differences in bioelectrical variables and physical performance tests between males and females for normally distributed variables. For non-normally distributed variables, the Mann-Whitney *U* test was used. Pearson's correlation coefficient was utilized to assess the linear correlation between bioelectrical variables and physical performance tests in males and females. The magnitude of correlations was interpreted as follows: r = 0.00–0.09, negligible; r = 0.10–0.39, weak; r = 0.40–0.69, moderate; r = 0.70–0.89, strong; r = 0.90–1.00, very strong [23]. When data were not normally distributed or the correlation was not linear, Spearman's rank correlation coefficient (Spearman's Rho) was used instead of Pearson's. To compare correlation coefficients between whole body and regional Bioelectrical Impedance Vector Analysis (BIVA) with physical performance tests, as well as between males and females, Fisher's r-to-z transformation was applied. Subsequently, comparisons for correlation coefficients with a standard variable [24] and independent correlation coefficients [25] were applied, respectively. An a priori power analysis was performed to determine the required sample size for this study. Given the anticipated large effect size in the comparison between males and females in athletic performance tests, the power analysis was conducted using G*Power 3.1.9.4 software with the following parameters: Effect size (Cohen's d) = 0.8 (large effect size), Alpha level = 0.05 (one-tailed), and Power (1-β) = 0.80. The results indicated that a minimum of 21 participants per group would be sufficient to detect a statistically significant difference. Regarding the correlation between bioelectrical impedance values and athletic performance, a one-tailed test was considered appropriate. An a priori power analysis using Pearson's correlation coefficient anticipated a medium effect size with the following parameters: Effect size (r) = 0.5 (medium effect size), Alpha level = 0.05 (one-tailed), and Power (1-β) = 0.80. The results indicated that a minimum of 23 participants would be necessary to detect a medium effect size.

## 3 Results

Descriptive statistics for the physical performance tests and BIVA results are presented in Table 1, Table 2, respectively. Significant differences were observed between sexes in both strength/speed tests and body composition analysis using raw bioelectrical data. Specifically, male athletes exhibited higher values in strength, as demonstrated by horizontal and vertical jumps, and speed, indicated by the five and 10-m sprints, compared to female athletes. Regarding raw bioelectrical data, males showed higher values for Phase Angle (PhA) and Levi Muscle Index (LMI), while females exhibited higher values for the ratios R/H and Xc/H.

**Table 1:** Results obtained from strength and speed performance tests by track and field athletes. Data are expressed as mean ± st. dev.

		Females (n = 31)	Males (n = 30)	p-value	d Cohen
HORIZONTAL JUMPS	Standing long jump (m)	2.1 ± 0.3	2.6 ± 0.3	<0.0001	−1.67
	Triple jump (m)	6.0 ± 1.0	7.4 ± 0.7	<0.0001	−1.61
SPRINT	5 m (sec)	1.3 ± 0.1	1.1 ± 0.1	<0.0001	−2.00
	10 m (sec)	2.1 ± 0.2	1.9 ± 0.1	<0.0001	−1.14
	10 m launched (sec)	1.5 ± 0.1	1.3 ± 0.1	<0.0001	−2.00
SQUAT JUMP	Height (cm)	26.5 ± 6.0	36.3 ± 7.2	<0.0001	−1.51
	Take-off force (kN)	0.7 ± 0.2	1.1 ± 0.3	<0.0001	−1.48
	Landing force (kN)	1.1 ± 0.4	1.6 ± 0.5	<0.0001	−1.08
	Maximum concentric power (kW)	2.5 ± 0.8	4.1 ± 1.2	<0.0001	−1.53
	Average speed concentric phase (m/s)	1.0 ± 0.4	1.0 ± 0.5	0.7095	0.00
	Peak speed (m/s)	2.4 ± 0.4	2.9 ± 0.4	<0.0001	−1.25
	Take-off speed (m/s)	2.3 ± 0.4	2.8 ± 0.4	<0.0001	−1.25
COUNTER MOVEMENT JUMP	Height (cm)	28.8 ± 7.5	39.9 ± 8.2	<0.0001	−1.41
	Take-off force (kN)	0.6 ± 0.2	0.9 ± 0.2	<0.0001	−1.50
	Landing force (kN)	1.0 ± 0.4	1.6 ± 0.5	<0.0001	−1.29
	Maximum concentric power (kW)	2.5 ± 0.7	4.1 ± 1.2	<0.0001	−1.58
	Average speed concentric phase (m/s)	1.4 ± 0.2	1.7 ± 0.3	0.0003	−1.16
	Peak speed (m/s)	2.5 ± 0.4	3.0 ± 0.3	<0.0001	−1.37
	Take-off speed (m/s)	2.4 ± 0.4	2.9 ± 0.3	<0.0001	−1.41
STIFFNESS TEST	Height (cm)	24.5 ± 6.5	32.8 ± 6.6	<0.0001	−1.26
	Take-off force (kN)	2.2 ± 0.4	3.0 ± 0.7	<0.0001	−1.33
	Landing force (kN)	2.3 ± 0.5	3.2 ± 0.8	<0.0001	−1.26
	Maximum concentric power (kW)	3.6 ± 1.0	5.8 ± 1.5	<0.0001	−1.70
	Average speed concentric phase (m/s)	1.6 ± 0.2	1.9 ± 0.2	<0.0001	−1.50
	Peak speed (m/s)	2.4 ± 0.3	2.9 ± 0.3	<0.0001	−1.67
	Take-off speed (m/s)	2.4 ± 0.3	2.9 ± 0.3	<0.0001	−1.67

**Table 2:** In summary, td from whole body and regional BIVA in track and field athletes. Data are expressed as mean ± st. dev. BIVA: Bioelectrical Impedance Vector Analysis; H: Height; LMI: Levi Muscle Index; PhA: Phase Angle; R: Resistance; Xc: Reactance.

		Females (n = 31)	Males (n = 30)	p-value	d Cohen
Whole body BIVA	R (Ω)	575.0 ± 66.3	479.2 ± 65.2	<0.0001	1.46
	R/H (Ω/H)	346.9 ± 45.0	266.2 ± 37.5	<0.0001	1.92
	Xc (Ω)	65.8 ± 7.3	61.0 ± 6.8	0.0090	0.68
	Xc/H (Ω/H)	39.7 ± 5.0	33.9 ± 4.0	<0.0001	1.29
	PhA (°)	6.6 ± 0.4	7.3 ± 0.6	<0.0001	1.31
	LMI	1.9 ± 0.3	2.8 ± 0.6	<0.0001	1.88
Regional BIVA Lower limb	R (Ω)	499.4 ± 65.3	434.0 ± 59.0	0.0001	1.03
	R/H (Ω/H)	301.1 ± 41.7	241.0 ± 33.4	<0.0001	1.57
	Xc (Ω)	62.1 ± 7.8	59.0 ± 7.4	0.1214	0.41
	Xc/H (Ω/H)	37.4 ± 5.1	32.8 ± 4.3	0.0003	0.98
	PhA (°)	7.1 ± 0.7	7.8 ± 0.6	0.0002	1.07
	LMI	2.4 ± 0.4	3.3 ± 0.6	<0.0001	1.78

Table 3, Table 4 present the correlations between bioelectric variables and physical performance tests for females and males, respectively. In females, weak to moderate negative correlations were observed for R/H (ranging from r = −0.38 to r = −0.54), while weak to moderate positive correlations were found for PhA (ranging from r = 0.36 to r = 0.59) and LMI (ranging from r = 0.36 to r = 0.69), both in whole body and regional BIVA values. For males, moderate to strong correlations were noted, with negative correlations observed for R/H (ranging from r = −0.40 to r = −0.72) and Xc/H (ranging from r = −0.27 to r = −0.67), and positive correlations observed for PhA (ranging from r = 0.29 to r = 0.75) and LMI (ranging from r = 0.37 to r = 0.80), across most variables.

**Table 3:** The correlation matrix between strength and speed tests with raw bioelectrical data from female track and field athletes' whole-body and regional BIVA approaches. Spearman's Rho correlations are reported in bold.

		Whole body BIVA				Regional BIVA			
		R/H	XC/H	PHA	LMI	R/H	XC/H	PHA	LMI
HORIZONTAL JUMP	Standing long jump	**−0.15**	**0.05**	**0.46**^**§**^	0.36*	**−0.16**	**0.15**	**0.52**^**§**^	0.44*
	Triple jump	**−0.33**	**0.10**	**0.49**^**§**^	**0.51**^**§**^	**−0.30**	**0.02**	**0.53**^**§**^	**0.52**^**§**^
SPRINT	5 m	**0.22**	**0.05**	**−0.30**	−0.38*	**0.16**	**0.10**	**−0.17**	**−0.24**
	10 m	**0.01**	**−0.01**	**−0.37**	**−0.31**	**0.11**	**−0.00**	**−0.27**	**−0.25**
	10 m launched	**0.11**	**−0.12**	**−0.50**^**§**^	**−0.35**	**0.23**	**−0.15**	**−0.52**^**§**^	**−0.46**^**§**^
SQUAT JUMP	Height	**−0.22**	−0.09	**0.34**	**0.32**	**−0.22**	0.01	**0.46**^**§**^	0.48^§^
	Take off force	−0.32	−0.08	**0.53**^**§**^	**0.52**^**§**^	**−0.33**	−0.04	**0.48**^**§**^	**0.55**^**§**^
	Landing force	−0.21	−0.20	0.08	0.24	**−0.03**	−0.24	−0.14	0.06
	Maximum concentric power	−0.42*	−0.18	0.41*	0.53^§^	**−0.30**	−0.08	0.43*	0.53^§^
	Average speed concentric phase	**−0.13**	**−0.13**	**0.06**	**0.06**	**−0.23**	**−0.12**	**0.17**	**0.18**
	Peak speed	**−0.33**	**−0.15**	**0.24**	**0.34**	**−0.28**	**−0.01**	0.37*	0.40*
	Take-off speed	**−0.33**	**−0.13**	**0.26**	**0.35**	**−0.28**	**0.003**	0.39*	0.41*
COUNTER MOVEMENT JUMP	Height	−0.18	0.01	0.37*	0.27	**−0.14**	0.09	**0.49**^**§**^	0.38*
	Take off force	−0.12	0.05	0.37*	0.31	**0.04**	0.07	0.20	0.20
	Landing force	**−0.29**	**−0.13**	**0.24**	**0.27**	−0.38*	**−0.09**	**0.28**	0.38*
	Maximum concentric power	−0.22	−0.05	0.36*	0.34	**−0.18**	0.02	0.40*	0.38*
	Average speed concentric phase	−0.06	0.11	0.31	0.17	**−0.13**	0.16	**0.56**^**ç**^	0.37*
	Peak speed	−0.03	0.13	0.31	0.14	**−0.04**	0.18	**0.42**^**§**^	0.38*
	Take-off speed	−0.03	0.14	0.32	0.15	**−0.03**	0.20	0.42*	0.38*
STIFFNESS TEST	Height	**−0.33**	−0.21	0.34	0.37*	**−0.27**	−0.11	0.33	0.40*
	Take off force	−0.51^§^	−0.29	0.42*	0.59^ç^	**−0.51**^**§**^	−0.19	0.48^§^	0.66 ^ç^
	Landing force	−0.49^§^	−0.27	0.47^§^	0.59^ç^	**−0.54**^**§**^	−0.19	0.49^§^	0.69 ^ç^
	Maximum concentric power	−0.42*	−0.22	0.36*	0.50^§^	−0.43*	−0.11	0.48^§^	0.60 ^ç^
	Average speed concentric phase	**−0.33**	−0.23	0.29	0.38*	**−0.28**	−0.14	0.29	0.37*
	Peak speed	**−0.35**	−0.19	**0.35**	0.42*	**−0.28**	−0.09	0.37*	0.41*
	Take-off speed	−0.30	−0.11	**0.34**	0.38*	**−0.22**	−0.01	0.38*	0.38*

Significance: *, *p* < 0.05; §, *p* < 0.01; ç, *p* < 0.001.

BIVA: Bioelectrsssical Impedance Vector Analysis; H: Height; LMI: Levi Muscle Index; PhA: Phase Angle; R: Resistance; Xc: Reactance.

**Table 4:** The correlation matrix between strength and speed tests with raw bioelectrical data from male track and field athletes' whole-body and regional BIVA approaches. Spearman's Rho correlations are reported in bold.

		Whole body BIVA				Regional BIVA			
		R/H	XC/H	PHA	LMI	R/H	XC/H	PHA	LMI
HORIZONTAL JUMP	Standing long jump	−0.61^ç^	**−0.34**^**§**^	0.74^ç^	0.68^ç^	**−0.53**^**ç**^	**−0.20**	0.70^ç^	0.70^ç^
	Triple jump	−0.65^ç^	**−0.41**^**§**^	0.69^ç^	0.67^ç^	**−0.57**^**ç**^	**−0.25**	**0.64**^**ç**^	0.68^ç^
SPRINT	5m	**0.40**^**ç**^	0.28*	**−0.45**^**ç**^	**−0.47**^**ç**^	**0.33**^**§**^	**0.22**	**−0.38**^**§**^	**−0.40**^**§**^
	10m	**0.53**^**ç**^	**0.37**^**§**^	**−0.58**^**ç**^	**−0.61**^**ç**^	**0.47**^**ç**^	0.27*	**−0.51**^**ç**^	**−0.55**^**ç**^
	10m launched	**0.59**^**ç**^	**0.34**^**§**^	**−0.69**^**ç**^	**−0.69**^**ç**^	**0.56**^**ç**^	**0.19**	**−0.66**^**ç**^	**−0.68**^**ç**^
SQUAT JUMP	Height	−0.59^ç^	**−0.35**^**§**^	0.71^ç^	0.66^ç^	**−0.54**^**ç**^	**−0.19**	0.68^ç^	0.68^ç^
	Take off force	**−0.66**^**ç**^	**−0.42**^**ç**^	0.75^ç^	**0.74**^**ç**^	**−0.61**^**ç**^	−0.29*	0.64^ç^	0.73^ç^
	Landing force	**−0.49**^**ç**^	**−0.46**^**ç**^	0.31*	**0.47**^**ç**^	**−0.40**^**ç**^	**−0.40**^**§**^	0.20	**0.37**^**§**^
	Maximum concentric power	−0.68^ç^	**−0.49**^**ç**^	0.75^ç^	**0.75**^**ç**^	−0.61^ç^	**−0.35**^**§**^	0.62^ç^	0.73^ç^
	Average speed concentric phase	**−0.01**	**−0.01**	**0.01**	**0.03**	**−0.05**	**−0.01**	**0.08**	**0.08**
	Peak speed	**−0.53**^**ç**^	**−0.33**^**§**^	0.58^ç^	**0.58**^**ç**^	**−0.48**^**ç**^	**−0.19**	0.57^ç^	0.53^ç^
	Take-off speed	**−0.52**^**ç**^	−0.30*	0.60^ç^	**0.59**^**ç**^	**−0.48**^**ç**^	**−0.17**	**0.58**^**ç**^	0.55^ç^
COUNTER MOVEMENT JUMP	Height	−0.55^ç^	−0.31*	0.69^ç^	0.63^ç^	**−0.52**^**ç**^	**−0.16**	0.72^ç^	0.68^ç^
	Take off force	−0.58^ç^	**−0.41**^**ç**^	0.69^ç^	**0.69**^**ç**^	**−0.53**^**ç**^	−0.30*	**0.57**^**ç**^	**0.62**^**ç**^
	Landing force	**−0.48**^**ç**^	**−0.41**^**ç**^	0.35*	**0.47**^**ç**^	**−0.47**^**ç**^	**−0.35**^**§**^	0.29*	**0.46**^**ç**^
	Maximum concentric power	**−0.70**^**ç**^	**−0.49**^**ç**^	**0.70**^**ç**^	**0.76**^**ç**^	**−0.65**^**ç**^	**−0.35**^**§**^	**0.63**^**ç**^	**0.73**^**ç**^
	Average speed concentric phase	−0.45^ç^	−0.27*	0.63^ç^	0.59^ç^	**−0.43**^**ç**^	**−0.12**	0.64^ç^	**0.55**^**ç**^
	Peak speed	**−0.52**^**ç**^	−0.31*	0.60^ç^	**0.59**^**ç**^	**−0.47**^**ç**^	**−0.17**	**0.58**^**ç**^	**0.59**^**ç**^
	Take-off speed	**−0.52**^**ç**^	−0.31*	0.65^ç^	**0.60**^**ç**^	**−0.47**^**ç**^	**−0.16**	**0.61**^**ç**^	0.64^ç^
STIFFNESS TEST	Height	−0.56^ç^	**−0.39**^**§**^	**0.54**^**ç**^	**0.59**^**ç**^	**−0.46**^**ç**^	**−0.21**	**0.52**^**ç**^	**0.54**^**ç**^
	Take off force	−0.70^ç^	**−0.54**^**ç**^	**0.63**^**ç**^	0.78^ç^	−0.67^ç^	**−0.42**^**ç**^	0.62^ç^	0.77^ç^
	Landing force	−0.69^ç^	**−0.51**^**ç**^	**0.65**^**ç**^	0.77^ç^	−0.66^ç^	**−0.40**^**§**^	0.61^ç^	0.76^ç^
	Maximum concentric power	−0.72^ç^	**−0.56**^**ç**^	**0.66**^**ç**^	0.80^ç^	−0.67^ç^	**−0.41**^**ç**^	0.64^ç^	0.78^ç^
	Average speed concentric phase	**−0.55**^**ç**^	**−0.41**^**ç**^	**0.53**^**ç**^	**0.59**^**ç**^	**−0.46**^**ç**^	**−0.23**	**0.50**^**ç**^	**0.54**^**ç**^
	Peak speed	**−0.58**^**ç**^	**−0.42**^**ç**^	**0.57**^**ç**^	**0.63**^**ç**^	**−0.48**^**ç**^	**−0.24**	**0.52**^**ç**^	**0.57**^**ç**^
	Take-off speed	**−0.56**^**ç**^	**−0.41**^**ç**^	**0.56**^**ç**^	**0.61**^**ç**^	**−0.47**^**ç**^	**−0.23**	**0.52**^**ç**^	**0.56**^**ç**^

Significance: *, *p* < 0.05; §, *p* < 0.01; ç, *p* < 0.001.

BIVA: *Bioelectrical* Impedance Vector Analysis; H: Height; LMI: Levi Muscle Index; PhA: Phase Angle; R: Resistance; Xc: Reactance.

No significant differences were found between whole body and regional BIVA correlation coefficients with physical performance tests. However, male athletes exhibited statistically higher correlation coefficients compared to females in several instances.

- Squat jump maximum concentric power showed higher correlation with whole body PhA (p = 0.046, z = −1.99).
- Counter Movement Jump (CMJ) maximum concentric power exhibited higher correlation with regional body LMI (p = 0.050, z = −1.96).
- Stiffness test maximum concentric power demonstrated higher correlation with whole body LMI (p = 0.042, z = −2.04).

Additionally, there was a trend towards significance for the following correlations in males compared to females.

- Horizontal jump with whole body PhA (p = 0.093, z = −1.68) and whole body LMI (p = 0.094, z = −1.68).
- CMJ height (p = 0.088, z = −1.70), take-off force (p = 0.088, z = −1.70), and maximum concentric power (p = 0.069, z = −1.82) with whole body PhA.

## 4 Discussion

This study explores the relationship between body composition and physical performance, utilizing bioelectrical impedance analysis (BIA) to assess body tissues through traditional raw bioelectrical data such as R, Xc, and PhA, along with the newer parameter LMI to evaluate muscle mass. Additionally, the study investigates whether regional BIVA assessment offers more accurate insights compared to a whole-body approach, especially in sports emphasizing lower limb performance, such as track and field.

The findings of this study underscore the direct associations between body composition and physical performance. Consistent with previous research [[6], [7], [8]], R exhibited a negative correlation with performance, extending this relationship to strength and speed outcomes in competitive sports. Moreover, the study confirms the moderate to strong positive correlation between PhA, a parameter receiving considerable attention in the literature [[26], [27], [28]], and anaerobic performance among track and field athletes [29]. The inclusion of LMI in raw bioelectrical data also revealed a moderate to strong relationship within this context. It is noteworthy that while LMI has been validated in male athlete populations [4], our study provides initial insights into its relevance among female track and field athletes. These findings suggest a promising direction for future research to further validate and expand upon the role of LMI in assessing athletic performance in diverse athlete populations.

Using an accelerometer for vertical jump assessments in this study facilitated a more comprehensive analysis by integrating additional parameters. While the height value alone did not consistently provide sufficient performance information, maximum concentric power emerged as a crucial parameter for in-depth analysis.

Although the differences in body composition and physical performance between males and females have been well-documented in sports literature [30,31], we aimed to delve deeper into these distinctions by examining their relationship in this study. Following verification of sex-based differences, as detailed in Table 1, Table 2, the relationship between these variables was separately analyzed for males and females, as shown in Table 3, Table 4 The results reveal that male athletes exhibit statistically higher correlation values compared to females. This disparity may be attributed to bioelectrical impedance's reliance on water and lean tissues for conducting alternating currents, where fat mass serves as an insulator. Given that females generally possess higher physiological fat content, this factor can attenuate the correlation between raw bioelectrical data and strength/speed performance metrics, which are inherently influenced by muscle mass [32]. Supporting this hypothesis, the smallest differences in correlations between sexes were observed in the stiffness test, which predominantly engages the calf muscles, an area with lower fat content. The chosen performance tests focus on evaluating lower limb speed and strength. Consequently, individuals with greater anaerobic capacity, characterized by recruitment of a higher number of type II muscle fibers, are expected to achieve higher scores. This aspect likely contributes to the higher correlation values observed in male athletes, as type II muscle fibers have a larger cross-sectional area and therefore higher water content than type I fibers. Another plausible explanation is that female athletes may have lower strength levels compared to males, potentially due to lesser exposure to strength training throughout their athletic development. Evidence suggests that female teams typically undergo fewer weekly in-season strength and conditioning sessions compared to male teams [33]. Therefore, the reliability of test results in this study may be less robust for female athletes, resulting in lower correlation coefficients with bioelectrical variables.

The correlation between physical performance and whole-body versus regional BIVA is an ongoing area of study where evidence is still emerging regarding whether the regional approach provides superior information [16]. Direct comparisons between whole-body and regional approaches are sporadically reported [34]. Some studies suggest that regional BIVA assessments may better reflect body composition changes following physical exertion in longitudinal studies rather than showing stronger associations with physical performance in cross-sectional designs compared to the whole-body approach [5]. The varying degrees of correlation observed between physical performance and whole-body or regional BIVA across different studies can be attributed to several factors. Firstly, the competitive level of the sample plays a crucial role; athletes at higher competitive levels typically undergo more frequent and intense training, enhancing overall body fitness rather than focusing solely on regional aspects. Secondly, the nature of the performance tests conducted is influential. Analytical tests such as handgrip for upper limbs or seated knee extensions for lower limbs may show stronger correlations with regional evaluations. Conversely, tests requiring greater coordination, such as jumping and running tests, may benefit more from a whole-body approach. Moreover, the specific sport disciplines studied thus far are limited, with some studies including university students without specifying their sporting backgrounds. Thirdly, the sex of the study sample also influences the results. It is hypothesized that the greater lean mass in males modulates the correlation between bioelectrical variables and physical performance.

In our study involving track and field athletes at least at level 3 (Highly Trained/National Level), differences between whole-body and regional BIVA approaches may be explained by sex differences. Specifically, LMI and PhA derived from regional BIVA provided additional insights into squat jump and countermovement jump tests for female athletes. Conversely, these insights were less pronounced in male track and field athletes due to their greater upper limb muscle mass, which contributes more significantly to whole-body BIVA correlations with physical performance.

No significant differences in the degree of correlation between physical performance and PhA or LMI were observed between whole-body and regional approaches. These findings suggest that LMI can be considered a valuable raw bioelectrical data point with significant relationships to physical functionality, similar to PhA, which has been validated as an indicator of cellular functionality in both athletic and clinical populations [35,36]. Future research could explore the correlation between LMI and physical performance in non-sporting populations. However, neither LMI nor PhA demonstrated a higher predictive value for physical performance compared to each other. It is hypothesized that bioelectric parameters only partially explain physical performance and provide moderate relationships because they do not account for the coordinative and neuromotor aspects of motor tasks. Therefore, integrating LMI into evaluation processes may represent a more comprehensive approach.

This study possesses several strengths. Firstly, it is the first of its kind to investigate body composition using both whole-body and regional BIVA approaches within a track and field sports population. Secondly, the utilization of raw bioelectrical data without reliance on predictive equations minimizes potential errors associated with estimation calculations. Thirdly, the sample size aligns with similar studies combining body composition assessments and physical performance tests, ensuring statistical robustness. Finally, all assessments were conducted consistently using the same instrumentation and operator, enhancing the reliability and consistency of the results.

Despite its strengths, this study also has several limitations. Firstly, the study participants are from a single country, which may restrict the generalizability of findings to a broader global population of track and field athletes. Secondly, the athletes included in the study belong to at least level 3 (Highly Trained/National Level), limiting the applicability of study conclusions to athletes at different training levels and states. Thirdly, while Levi Muscle Index (LMI) has been validated primarily in male populations, its application in this study represents its first use in a female population, suggesting caution in interpreting its findings.

## 5 Conclusion

This study offers novel insights into the relationship between body composition and physical performance among athletes. Significant differences in the correlation between bioelectrical data and physical performance were observed based on sex, potentially influenced by higher fat content in females. The study also investigated the efficacy of regional versus whole-body BIVA approaches. However, a definitive conclusion regarding which approach provides superior information correlating with physical performance remains elusive.

Furthermore, the study identified that the level of competition and the nature of performance tests significantly impact the correlation between physical performance and BIVA measurements. Both Levi Muscle Index (LMI) and Phase Angle (PhA) emerged as valuable indicators of physical functionality. However, their predictive value for physical performance outcomes did not decisively favor one over the other.

In summary, this study underscores the complexity of the relationship between body composition and physical performance, highlighting the necessity for further research in this area.

## Practical applications

● Sex-Specific Evaluation: Differences in body composition adaptations and physical performance between sexes underscore the importance of evaluating the relationship between physical performance and body composition based on the athlete's sex.
● Bioelectrical Impedance Approach: The study suggests a preliminary preference for using whole-body bioelectrical impedance in initial analyses for track and field athletes, over the regional approach. This approach may provide higher correlations with physical performance metrics, particularly in tests emphasizing coordination and neuromotor aspects.
● Regional Approach in Male Athletes: For male athletes, especially in interpreting parameters like Phase Angle and Levi Muscle Index, the regional bioelectrical impedance approach may offer enhanced insights due to its ability to capture specific regional muscle characteristics.

## Data availability statement

The data supporting this study's findings are available in Mascherini, Gabriele; Levi Micheli, Matteo (2024), “Track and Field & BIVA”, Mendeley Data, V1, https://doi.org/10.17632/ttnpgykg39.1.

## Disclosure statement

The authors report there are no competing interests to declare.

## Funding details

This study has received no funding

## Ethics approval statement

The study was carried out in conformity with the ethical standards in the 1975 Declaration of Helsinki. Ethical approval for this study was granted by the Ethics Committee for Clinical Sport Research of Catalonia (Ethical Approval Code: 0022/CEICGC/2023).

## CRediT authorship contribution statement

**Gabriele Mascherini:** Writing – original draft, Methodology, Conceptualization. **Matteo Levi Micheli:** Methodology, Investigation, Data curation. **Sofia Serafini:** Methodology, Formal analysis, Data curation. **Claudia Politi:** Investigation, Data curation. **Eva Bianchi:** Investigation, Data curation. **Álex Cebrián-Ponce:** Writing – original draft, Data curation. **Marta Carrasco-Marginet:** Visualization, Validation. **Pascal Izzicupo:** Writing – original draft, Methodology, Conceptualization.

## Declaration of competing interest

The authors declare that they have no known competing financial interests or personal relationships that could have appeared to influence the work reported in this paper.
